# Spatiotemporal Cadence of Macrophage Polarisation in a Model of Light-Induced Retinal Degeneration

**DOI:** 10.1371/journal.pone.0143952

**Published:** 2015-12-02

**Authors:** Haihan Jiao, Riccardo Natoli, Krisztina Valter, Jan M. Provis, Matt Rutar

**Affiliations:** 1 John Curtin School of Medical Research, The Australian National University, Canberra, Australian Capital Territory, Australia; 2 ANU Medical School, The Australian National University, Canberra, Australian Capital Territory, Australia; University of Florida, UNITED STATES

## Abstract

**Background:**

The recruitment of macrophages accompanies almost every pathogenic state of the retina, and their excessive activation in the subretinal space is thought to contribute to the progression of diseases including age-related macular degeneration. Previously, we have shown that macrophages aggregate in the outer retina following damage elicited by photo-oxidative stress, and that inhibition of their recruitment reduces photoreceptor death. Here, we look for functional insight into macrophage activity in this model through the spatiotemporal interplay of macrophage polarisation over the course of degeneration.

**Methods:**

Rats were exposed to 1000 lux light damage (LD) for 24hrs, with some left to recover for 3 and 7 days post-exposure. Expression and localisation of M1- and M2- macrophage markers was investigated in light-damaged retinas using qPCR, ELISA, flow cytometry, and immunohistochemistry.

**Results:**

Expression of M1- (*Ccl3*, *Il-6*, *Il-12*, *Il-1β*, *TNFα*) and M2- (*CD206*, *Arg1*, *Igf1*, *Lyve1*, *Clec7a*) related markers followed discrete profiles following light damage; up-regulation of M1 genes peaked at the early phase of cell death, while M2 genes generally exhibited more prolonged increases during the chronic phase. Moreover, Il-1β and CD206 labelled accumulations of microglia/macrophages which differed in their morphological, temporal, and spatial characteristics following light damage.

**Conclusions:**

The data illustrate a dynamic shift in macrophage polarisation following light damage through a broad swathe of M1 and M2 markers. Pro-inflammatory M1 activation appears to dominate the early phase of degeneration while M2 responses appear to more heavily mark the chronic post-exposure period. While M1/M2 polarisation represents two extremes amongst a spectrum of macrophage activity, knowledge of their predominance offers insight into functional consequences of macrophage activity over the course of damage, which may inform the spatiotemporal employment of therapeutics in retinal disease.

## Introduction

Recruitment and activation of macrophages is prominent feature of virtually all pathogenic states of the retina. These responses are typically heterogeneous, and understood to comprise a conglomerate of resident and non-resident phagocytes. Microglial cells are glial constituents derived from the mononuclear phagocyte lineage, which serve as the primary resident macrophages of the retina. Using their motile processes, microglia persistently survey the retinal microenvironment, and facilitate homeostatic functions. During instances of retinal pathology, resident macrophage responses are supplemented by populations of bone-marrow derived macrophages recruited from the retinal and choroidal vasculature [1].

Microglia/macrophage infiltration (hereby referred to collectively as ‘macrophages’ for brevity) is well-characterised in a range of human dystrophies such as age-related macular degeneration (AMD) [2–6], retinitis pigmentosa [3], retinal detachment [7], glaucoma [8–10], and diabetic retinopathy [8,11]. Despite their beneficial homeostatic properties, excessive macrophage activation may result in damage to the neural environment. A number studies have shown that accumulation of macrophages in areas of retinal pathology exacerbates photoreceptor death, including models that mimic aspects of AMD pathology including laser-induced choroidal neovascularisation (CNV) [12], photo-oxidative damage [13–16], and carboxyethylpyrrole (CEP)-immunized mice [17], as well as retinal detachment [18], diabetic retinopathy [19,20], and glaucoma [21,22]. Knowledge of the exact means by which macrophages may mediate retinal degeneration are incomplete, though several lines of evidence point to their secretion of factors—such as the cytokines Il6, Il-1β and TNFα –which augment macrophage survival and negatively impact the retinal milieu ([17,23,24]).

Macrophage phenotypes may be broadly—but not exclusively—defined as ‘classically activated’ (M1 macrophages) or ‘alternatively activated’ (M2 macrophages). M1 macrophages are considered pro-inflammatory initiators, and are typified by high production of cytokines including Il-1β, and TNFα, as well as reactive nitrogen and oxygen intermediates. M2 macrophages, conversely, are commonly associated with tissue remodelling and repair through receptor/secretory augmentations which promote angiogenesis, efficient scavenging of debris, and resolution of inflammatory responses. Although M1/M2 states are recognised as a simplification of two extremes which form part of a spectrum of macrophage phenotypes, these are nevertheless acknowledged to offer broad insight into the functional consequences of macrophage activation in over the course of disease (reviewed in [25]).

We have shown previously—using a light-induced model of photo-oxidative damage and subretinal inflammation—that reducing local expression of the chemoattractant Ccl2 reduces retinal macrophage recruitment and photoreceptor death [26]. However, the protracted interplay between M1/M2 polarised macrophages in the post-light damage period has not been investigated. In the current study, we examine the broad spatiotemporal profile of M1- (*Ccl3*, *Il-6*, *Il-12*, *Il-1β*, *TNFα*) and M2- (*CD206*, *Arg1*, *Igf1*, *Lyve1*, *Clec7a*) markers in the light damage model, to better understand the roles these phenotypes play in retinal damage and recovery in the post-exposure period. We report a changing profile of recognized M1- and M2- related genes after exposure to bright light, indicating a transient burst of M1-mediated activity followed by increases in M2 gene expression during the recovery period. We also find that, Il-1β and CD206 markers spatiotemporally label distinct macrophage subsets comprising cells associated with the ONL/subretinal space and retinal vasculature, respectively.

## Methods

### Animals and light damage paradigm

All experiments conducted were in accordance with the ARVO Statement for Use of Animals in Ophthalmic and Vision Research; the study was approved by the Animal Experimentation Ethics Committee (AEEC) of the Australian National University (Ethics ID: A2014/56). Adult Sprague-Dawley (SD) rats were exposed 1000 lux of light damage (LD) for the period of 24 hrs, in accordance with a previous protocol [27]. Additionally, some animals were returned to dim-light (5 lux) conditions immediately following LD for a period of 3 or 7 days, to assess post-exposure changes. Food, water, and environmental enrichment were provided to the animals *ad libitum*, and their physical well-being over the experimental period was checked 4 times a day by experienced technicians and veterinarians.

### Tissue collection and processing of whole retinas

Animals were euthanized with an overdose of barbiturate administered via intraperitoneal injection (Valabarb; Virbac, NSW, Australia). The left eye from each animal was marked at the superior surface for orientation then enucleated and processed for cryosectioning. The whole retina from the right eye was excised through a corneal incision and prepared for either RNA or protein extraction.

Eyes for cryosectioning were immediately immersion fixed in 4% paraformaldehyde in 0.1M PBS for 4 hours at room temperature, then processed as previously described [28] and cryosectioned at 16 μm. Retinas for RNA extraction were immediately deposited in pre-chilled RNAlater solution (Thermo Fisher Scientific, Carlsbad, CA). RNA was then extracted from each sample following methodology established previously [29]. Retinas for protein extraction were promptly immersed in a chilled solution of Cellytic MT (Sigma-Aldrich, St Louis, MO) supplemented with a protease inhibitor cocktail (Sigma-Aldrich). Samples were homogenised via mechanical means, and then incubated for 10 minutes on ice. The resulting homogenate was centrifuged at 10000g for 10 minutes at 4°C; the supernatant was collected and stored at -70°C until use. The concentration the protein samples was determined using a Bradford assay (Cat#500–0205; Biorad, Hercules, CA), which was measured via absorbance at 595 nm using a TECAN Infinite 200 PRO (TECAN Seestrasse, Männedorf, Switzerland).

### Flow cytometry

Rats at each time point were euthanized as described in the previous section. Retinas from both eyes were promptly removed through a corneal incision. Retinas from each animal were pooled and immediately placed in chilled Hank's balanced salt solution (HBSS), and then subjected to light mechanical separation using a razor blade. Samples were transferred into 0.2% papain digestion cocktail as described in a previous protocol [30]. For permeabilisation, samples were then fixed in 4% paraformaldehyde for 10 minutes, washed with HBSS, and then resuspended in 0.1% saponin (Santa Cruz Biotechnology, Dallas, TX). The samples were then incubated in staining buffer containing the desired antibodies (Table 1) for 30 minutes at 4°C, then washed twice in HBBS and resuspended in staining buffer. Samples were run through a BD Fortessa flow cytometer (BD Biosciences, Franklin Lakes, NJ), from which 100,000 cells were sorted for each sample. Changes in the number CD11b, CD206, and Il-1β labelled cells over time were calculated as a percentage of the parent population of sorted cells.

**Table 1:** Antibodies used for flow cytometry.

Raised Against	Dilution	Source
**Primary**		
CD11b—PE	1:500	Cat# 201807; Biolegend, San Diego, CA
CD206	1:500	Cat# ab8918; Abcam, Cambridge, MA
IL-1β	1:100	Cat#; R&D Systems, Minneapolis, MN
**Secondary**		
Rabbit IgG—Alexa488	1:500	Cat# A21206; Thermo Fisher Scientific, Carlsbad, CA
Goat IgG—Biotin	1:200	Cat# SAB3700318; Sigma Aldrch, St. Louis, Missouri
Streptavidin—PE/Cy7	1:500	Cat#405206; Biolegend, San Diego, CA

### Polymerase chain reaction (PCR)

First-strand cDNA synthesis was performed as described previously [29]. Expression was measured using commercially available TaqMan hydrolysis probes (Thermo Fisher Scientific); the particulars are provided in Table 2. The hydrolysis probes were applied in the same fashion as our previous study [29]. Fold change was determined using the ΔΔC_q_ method where the expression of the target gene was normalised relative to the expression of the reference gene glyceraldehyde-3-phosphate dehydrogenase (*Gapdh*). *Gapdh* was deemed suitable since it does not alter in expression with respect retinal light damage, as indicated by several investigations [27,31,32].

**Table 2:** Taqman hydrolysis probes used for qPCR.

Gene Symbol	Gene Name	Catalogue	Entrez Gene ID
ARG1	Arginase	Rn00691090_m1	NM_017134.2
CCL3	Chemokine (C-C motif) ligand 3	Rn00564660_m1	NM_053858.1
IGF1	Insulin-like growth factor 1	Rn00710306_m1	NM_001082477.2
IL12B	Interleukin 12B	Rn00575112_m1	NM_022611.1
IL1B	Interleukin 1 beta	Rn00580432_m1	NM_031512.2
IL6	Interleukin 6	Rn01410330_m1	NM_012589.1
LYVE1	Lymphatic vessel endothelial hyaluronan receptor 1	Rn01510421_m1	NM_001106286.1
CD206 (MRC1)	Mannose receptor, C type 1	Rn01487342_m1	NM_001106123.1
TNF	Tumor necrosis factor	Rn00562055_m1	NM_012675.3
CLEC7A	C-type lectin domain family 7, member A	Rn01459401_m1	NM_001173386.1
GAPDH	Glyceraldehyde-3-phosphate dehydrogenase	Rn99999916_s1	NM_017008.3

### ELISA

The levels of Il-1β and CD206 in retinas following LD were quantified using sandwich ELISA kits, comprising of 96-well microplates coated with an antibody against Il1β (Cat# RLB00; R&D Systems, Minneapolis, MN) or CD206 (Cat# SEB542Ra; Cloud-Clone Corp, Houston, TX). The kits were used as per the manufacturer’s instructions, using protein samples obtained from whole-retina homogenates described in the earlier section. The staining intensity was determined spectrophotometrically at 450 nm using a TECAN Infinite 200 PRO (TECAN).

### Immunohistochemistry

Cryosections from each timepoint were used for immunohistochemical analysis, using primary antibodies for Il-1β (1:500, Cat# AF501; R&D Systems), CD206 (1:200, Cat# ab64693; Abcam, Cambridge, MA), and IBA1 (1:500, Cat# 019–19741; Wako, Osaka, Japan). Immunohistochemistry was performed using methodology previously described [29]. Immunofluorescence was viewed using a Nikon A1 confocal microscope, and acquired with Nikon NIS—Elements C Software.

### Analysis of cell death

TUNEL labelling was used to quantify photoreceptor apoptosis in cryosections, and utilised protocol which has been documented previously [33]. Counts were made of TUNEL positive cells in the outer nuclear layer (ONL), and were performed along the full-length of retinal sections cut in the vertical meridian (superio-inferior), including the optic disc. The final count from each animal is the average at comparable locations in 2 non-sequential sections.

### Statistical Analysis

All graphing and statistical analysis was performed using Prism 6 (GraphPad Software, CA, USA). A one-way analysis of variance (ANOVA), with Tukey’s multiple comparison post-test was applied where desired, and differences with a P value < 0.05 were considered statistically significant.

## Results

### Temporal expression of M1-M2 genes in the retina and their relation to light-induced photoreceptor death

**Fig 1:**
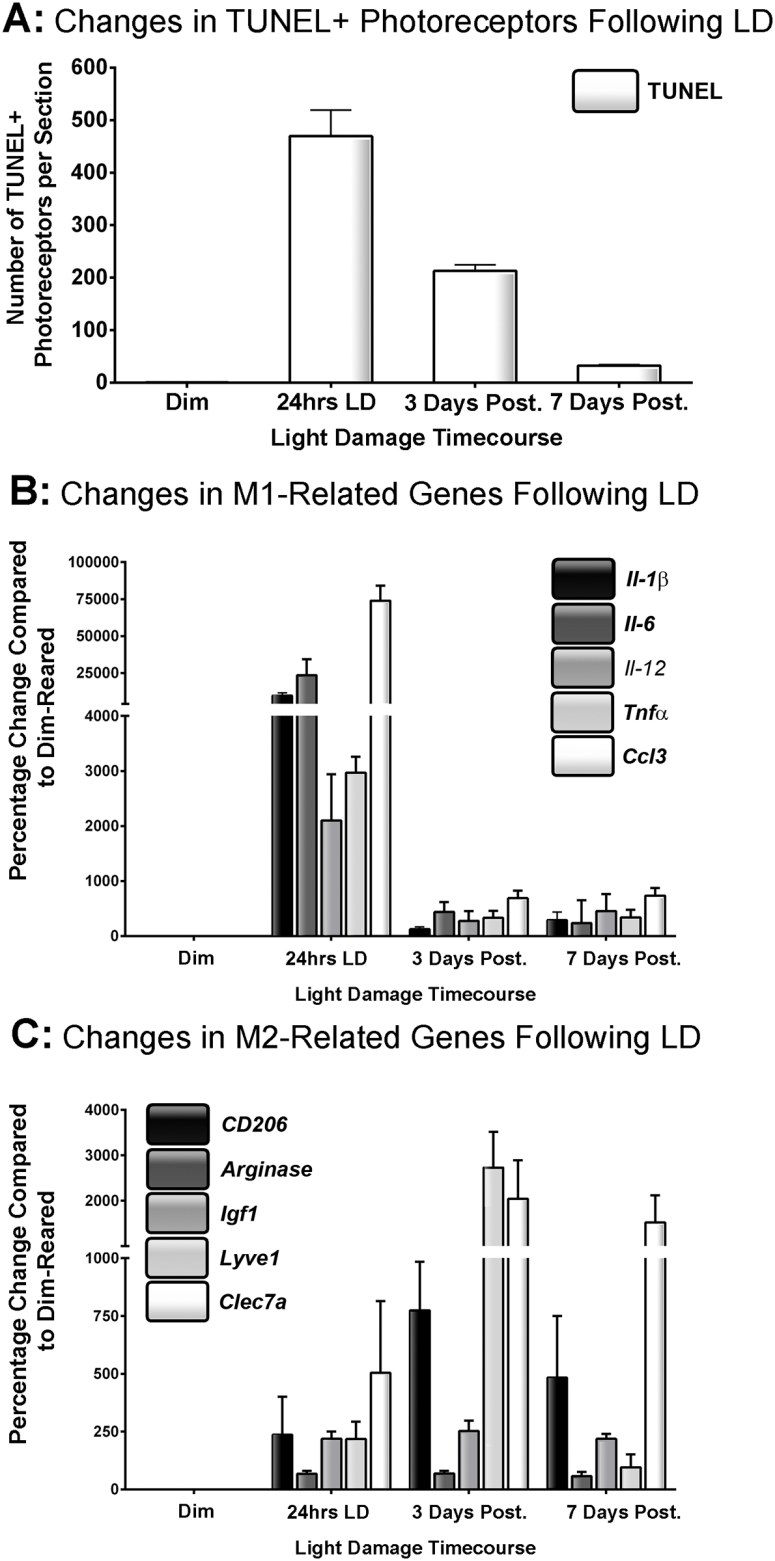
Expression of M1- and M2- associated markers via qPCR following LD. **A:** The number TUNEL+ photoreceptors increased profoundly after 24hrs LD. This number decreased progressively during the post-exposure period, though chronic levels of cell death remained after 7 days post-exposure. **B:** The expression of all M1 genes peaked at 24hrs LD, only to then diminish rapidly during the post-exposure period. Minimal up-regulation in these genes was observed after 7 days. **C:** M2-associated genes displayed more protracted up-regulation over the LD timecourse. Arginase and *Igf1* expression had modest yet consistent up-regulation over time course. Prolonged up-regulation was also evident for *CD206*, *Clec7a*, and *Lyve1* though with some temporal variation; *CD206*, *Clec7a* exhibited strong sustained up-regulation from 3 to 7 days post-exposure, while Lyve1 peaked at 3 days and was vastly reduced after 7. The overall trend in expression for each gene was significant by ANOVA (P < 0.05); N = 5 for each timepoint.

A large increase in the number of TUNEL+ photoreceptors was observed after 24hrs LD (Fig 1A), as reported previously [27]. By 3 and 7 days post-exposure, the number of TUNEL+ nuclei had decreased substantially. Expression of a range of M1- and M2- associated genes (as reviewed in [25,34]) was assessed over the same time course (Fig 1B–1C). Each of the M1-associated genes demonstrated peak expression (Fig 1B) immediately following 24hrs LD (P<0.05), when cell death was maximum. Up-regulation ranged from 23400% for *Il-6* to 9600% for *Il-1β* compared to dim-reared animals. At 3 days post-exposure, up-regulation of these genes was an order of magnitude lower (P<0.05), and remained at approximately the same levels at 7 days post exposure. In contrast, expression of M2-associated genes (Fig 1C) tended to be more highly expressed at the 3 day and 7 day timepoints, although there was some temporal variance between genes. Expression of Arginase and *Igf1* were up-regulated to levels that are consistent over the time course (~60% and 250%, respectively) compared to dim-reared controls. *CD206* and *Clec7a* were highly upregulated at 3 post-exposure (775% and 2000%, respectively), then somewhat decreased at 7 days (P>0.05); *Lyve1* was also highly up-regulated at 3 days (2700%, P<0.05), but significantly reduced by 7 days (95%, P<0.05). The trend in expression of all genes assessed was significant, as determined by one way ANOVA (P<0.05).

### Analysis of Il-1β and CD206 labeling among CD11b+ cells following light damage using flow cytometry

**Fig 2:**
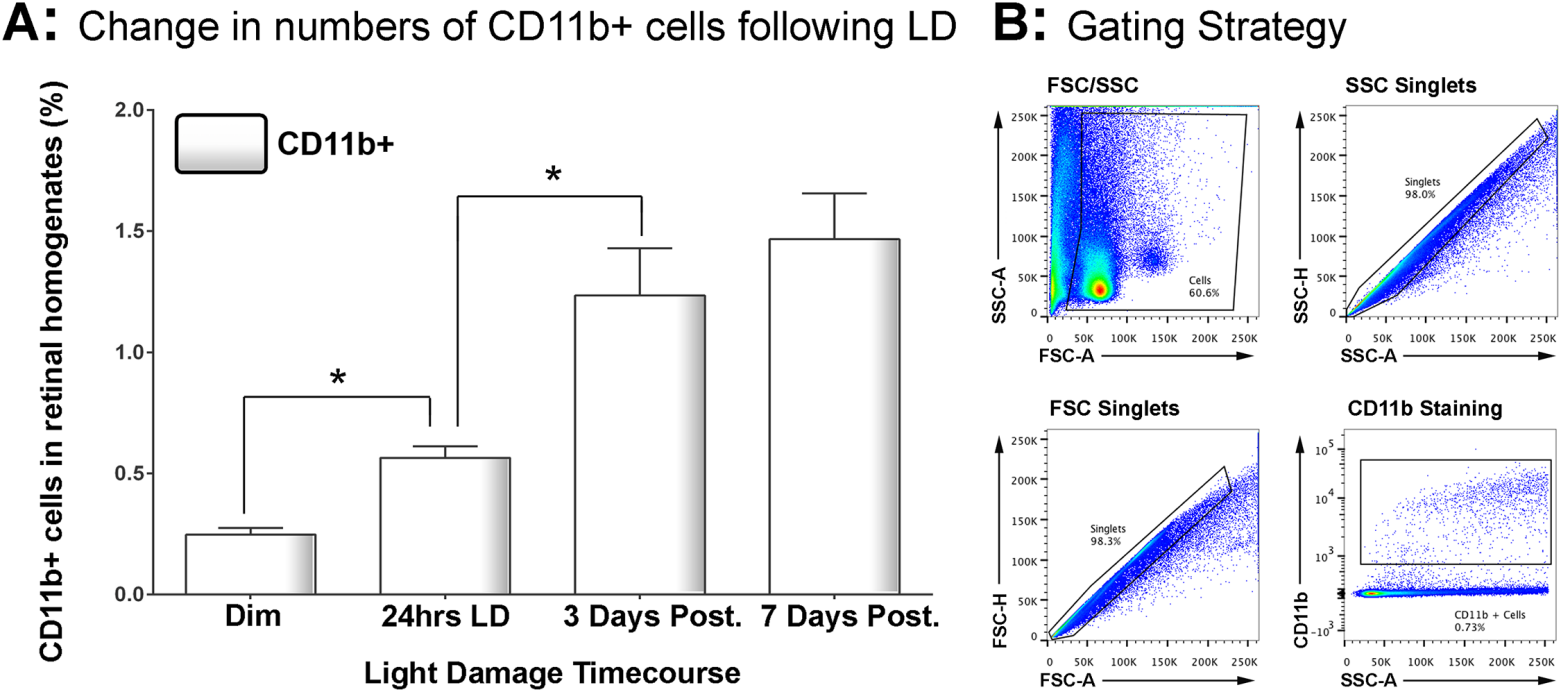
Changes the number retinal CD11b+ cells following LD. **A:** Using flow cytometry, the percentage of CD11b+ cells within the retinal homogenate was found to double following 24hrs LD (P < 0.05). The proportion of CD11b+ cells continued to increase after 3 days post-exposure (P < 0.05), through was stabilised by 7 days (P > 0.05). The overall trend in CD11b+ cell number across the time course was significant by ANOVA (P < 0.05); N = 5 for each timepoint. **B:** Representative flow cytometry plots with gating strategies are shown for a 3 days post-exposure sample stained for CD11b; gating methodology was applied equally for all samples.

**Fig 3:**
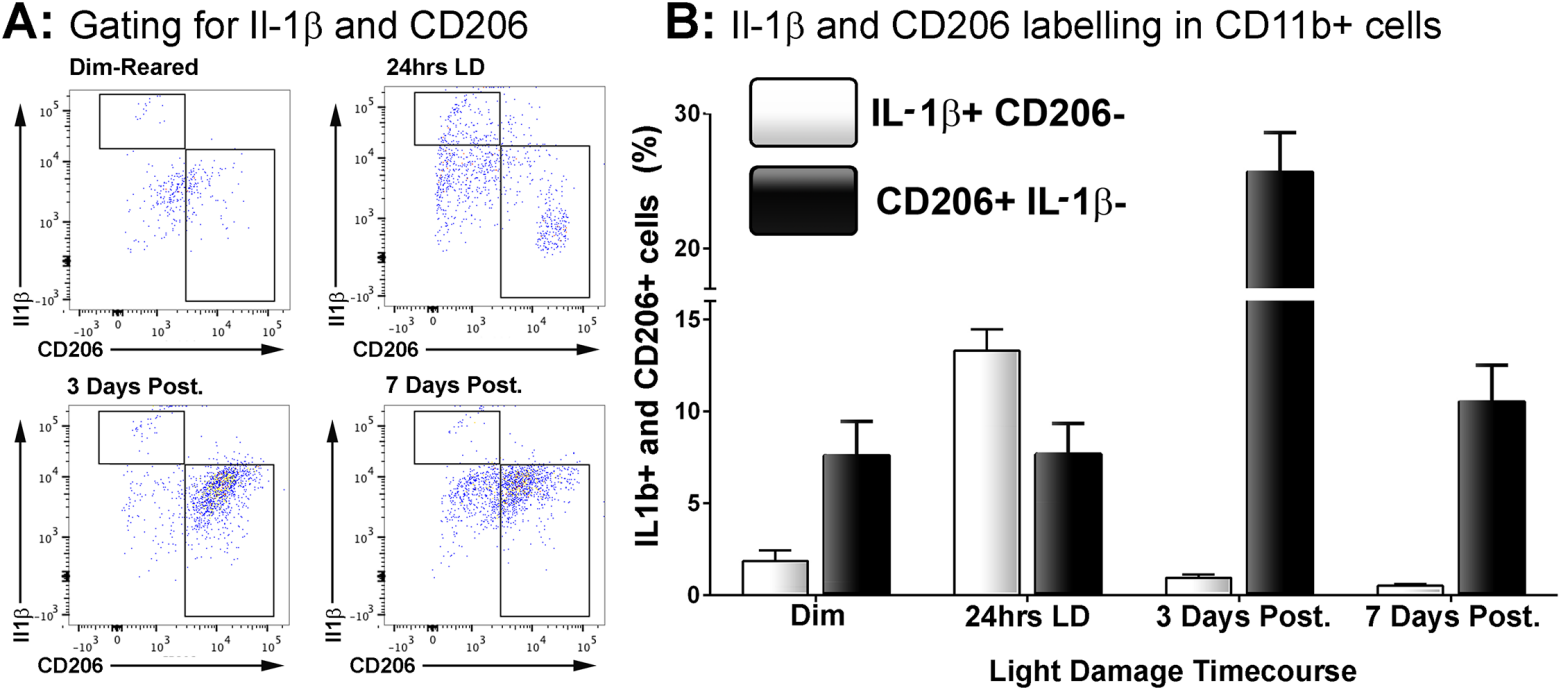
Correlation of CD206 and Il-1β immunolabelling within the CD11b+ macrophage population following LD. **A:** Representative flow cytometry plots examine CD206+ and Il1b+ cell counts within the CD11b population following light damage. For the most part, Il-1β and CD206 cells occupied mutually distinct subsets within the population CD11b cells. **B:** Quantification of Il-1β+/CD206- and CD206^+^/Il-1β- cells as percentage of the CD11b+ population following LD. There was a sharp increase in the proportion of Il-1β+/CD206- cells immediately following 24hrs LD (P<0.05), though this then decreased dramatically afterward and was similar to control samples by 7 days (P>0.05). For CD206+/Il-1β- cells, there was no change in their proportion at 24hrs LD (P>0.05). At 3 days post-exposure however the proportion of CD206+/Il-1β- cells had tripled (P<0.05), though this was then reduced to near control proportions by 7 days post-exposure (P<0.05). The trend of both Il-1β+/CD206- and CD206+/Il-1β- cells across the time course were significant by ANOVA (P < 0.05); N = 5 for each timepoint.

Il-1β and CD206 were selected (from genes in Fig 1) for further analysis due to their robust up-regulation and divergent differential expression. Changes in the number of CD11b+ macrophages in the retina following LD were determined using flow cytometry (Fig 2), and labelling for Il-1β and CD206 within this population was determined at 24 hrs light exposure, and after 3 and 7 days recovery (Fig 3). Representative gating strategies and scatter blots for CD11b^+^ cells are shown in Fig 2B. In dim-reared control retinas, the proportion of CD11b+ cells within retinal cell homogenates was approximately 0.24% (Fig 2A). After 24hrs LD, the retinal CD11b+ population had increased significantly to 0.56% (P < 0.05), and then further to 1.23% by 3 days post-exposure (P < 0.05). The population was found to have increased slightly more after 7 days (1.47%), although this was not statistically different from 3 days (P > 0.05).

Staining for CD206 and Il-1β in the population of CD11b+ macrophages suggests that these markers predominately label distinct subsets cells that are either Il1β+/CD206- or CD206+/Il-1β- (Fig 3A); a measure of Il1b+/CD206+ labelling was negligible as demonstrated in the flow cytometry plots (Fig 3A).

The changing proportions of Il-1β+/CD206- and CD206+/Il-1β- cells within the CD11b population over the timecourse is shown in Fig 3A–3B. Percentage of Il1ß+/CD206- macrophages increased from approximately 1.8% in controls to 13.3% at 24 hrs light damage (Fig 3B; P<0.05). However, this dramatic increase was followed by rapid return to proportions similar to controls 3 and 7 days post-exposure (0.5%; P>0.05). In contrast the percentage of CD206+/Il-1β- cells within the CD11b population did not change at 24hrs LD compared to controls (7.7% and 7.6%; P>0.05), although it was found to roughly triple at 3 days post-exposure (25.6%; P<0.05). After 7 days post-exposure however the proportion had dropped 10.5% (P<0.05), and while this was higher than their proportion in control samples it was not statistically significant (P>0.05)

### Spatiotemporal localization of Il1β+ and CD206+ macrophages in retinas following light damage

**Fig 4:**
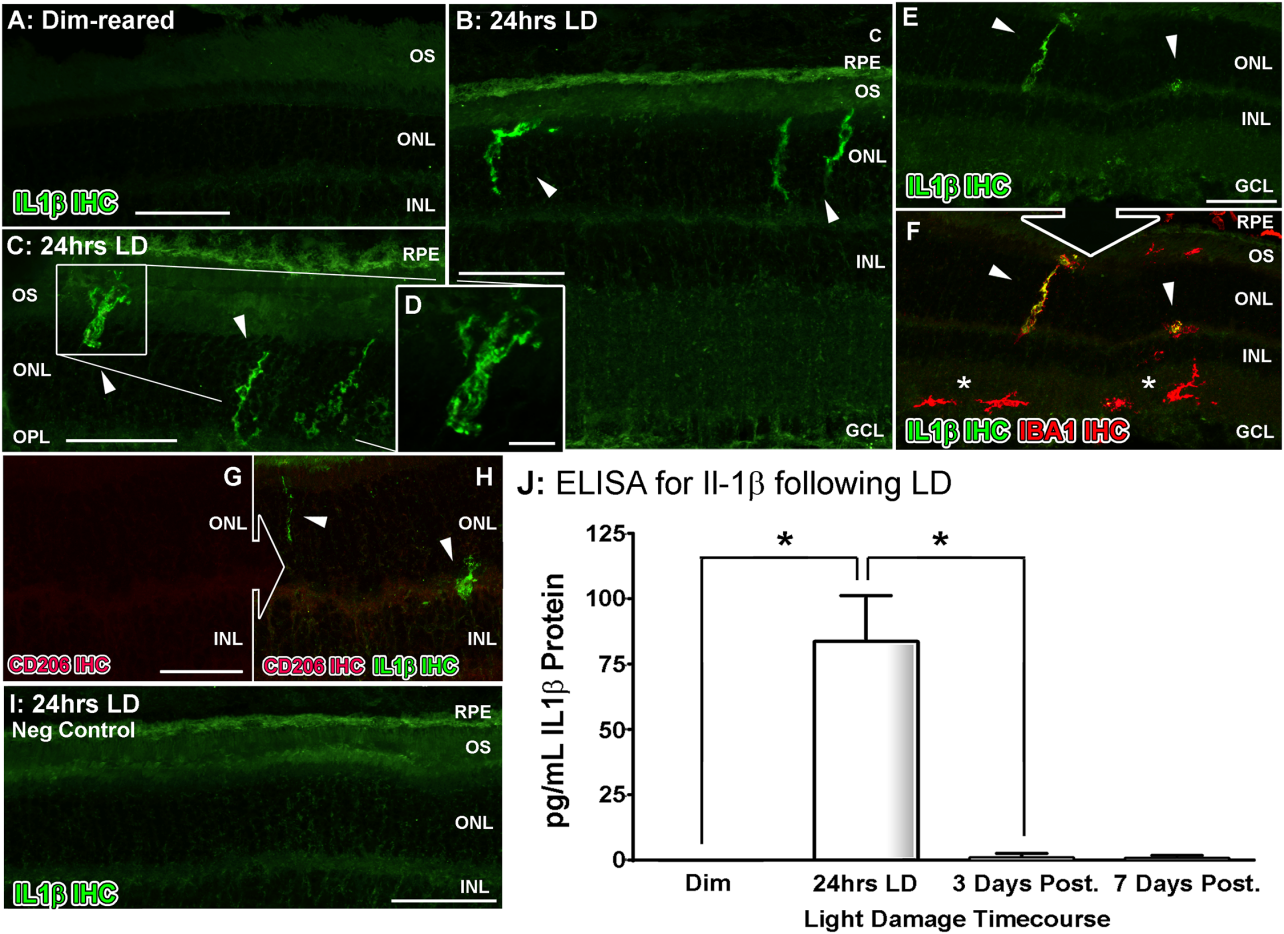
Spatiotemporal analysis of retinal Il-1β protein levels following LD. **A-G:** Immunohistochemical assessment of Il-1β expression (green) in retinal cryosections over the course of LD. **A:** Immunoreactivity (IR) for Il-1β protein was not observed in dim-reared animals. **B-D:** Il-1β-IR was present among ramified nuclei situated within the ONL and OS (arrowheads) immediately following exposure to 24hrs LD. **E-F:** Il-1β-IR co-localised with IBA1+ cells (red) situated in the ONL/OS, though was not apparent in IBA1+ cells outside the vicinity of the ONL (asterisks). **G-H:** Il-1β-expressing cells (H, arrowheads) did not show any discernible IR for the M2 marker CD206 (red). **I:** Negative control sections, in which the primary antibody was omitted, did not show any resemblance to the IR for Il-1β evidenced in C-D at 24hrs LD. **J:** ELISA for Il-1β protein indicated an increased abundance of the protein immediately after 24hrs LD (P<0.05), and which was virtually undetectable at all other time points. C, choroid; GCL, ganglion cell layer; INL, inner nuclear layer; IHC, immunohistochemistry; ONL, outer nuclear layer; OS, outer segments; RPE, retinal pigment epithelium. The trend in ELISA protein levels was significant by ANOVA (P < 0.05); N = 3 for each timepoint.

**Fig 5:**
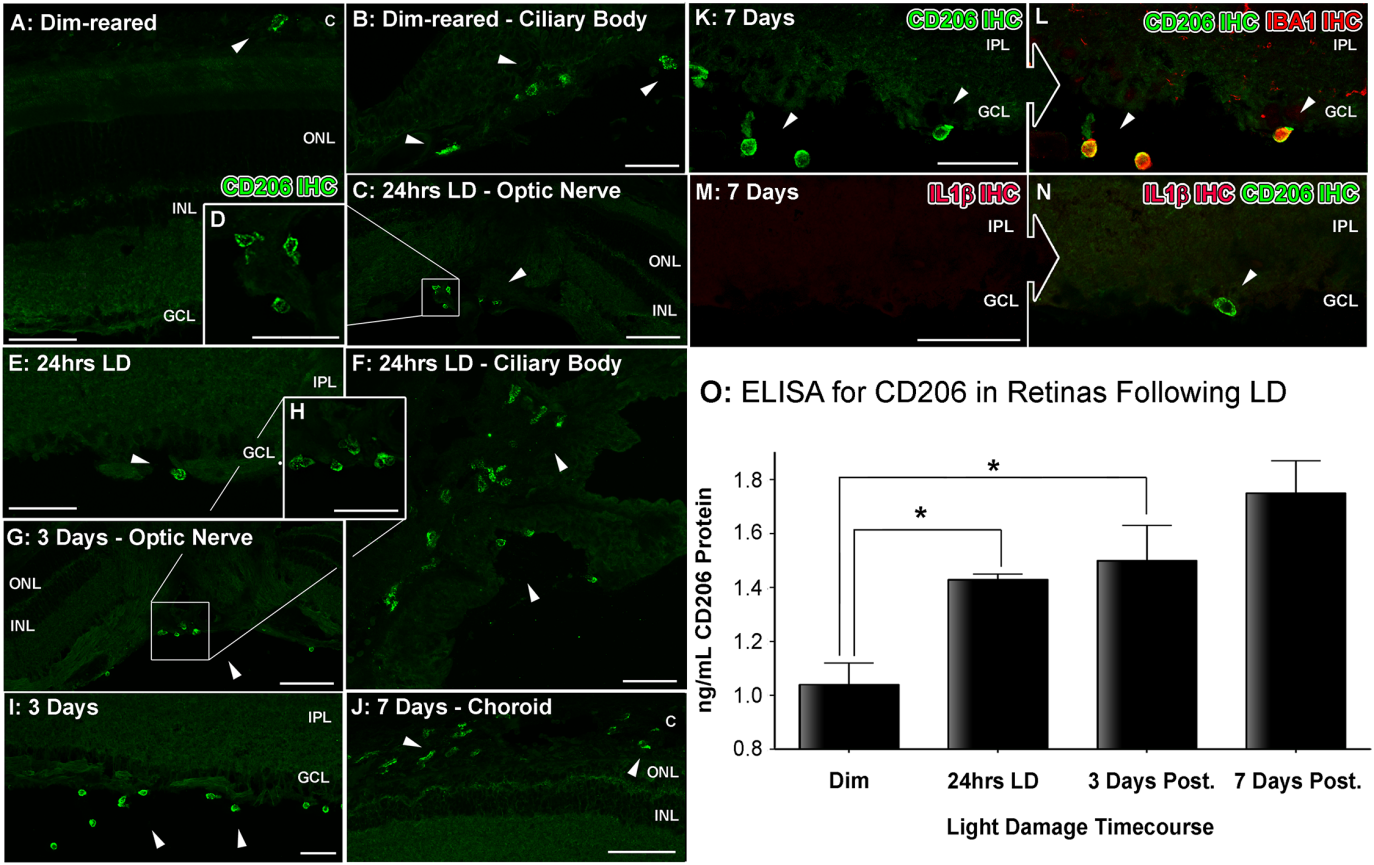
Spatiotemporal analysis of retinal CD206 protein levels following LD. **A-L:** investigation of CD206 immunoreactivity (IR, green) in retinal cryosections over the course of LD. **A-B:** In dim-reared animals, immunoreactivity (IR) for Il-1β protein was occasionally observed within nuclei (arrowheads) amongst the choroid (A) and ciliary body (B). **C-E:** Following 24hrs LD, CD206+ nuclei appeared from the ciliary body (C-D, arrowheads) and among the superficial retinal vasculature (E, arrowhead) **F:** At 24hrs LD, CD206+ cells were also more abundant within the ciliary body (arrowheads). **G-I:** There was increased abundance of CD206+ nuclei among optic nerve head (G-H) and superficial retinal vasculature (I) after 3 days post-exposure (arrowheads), compared to 24hrs LD. **J:** CD206+ cells were occasionally found accumulating within the choroid at 7 days post-exposure (arrowheads). **K-L:** All IR for CD206 was found to correlate with circular IBA1+ cells (red). **M-N:** CD206-expressing cells (N, arrowhead) did not show any detectable IR for the M1 marker Il-1β (red). **O:** Quantification of CD206 protein levels in retinas via ELISA. At 3 and 7 days post-exposure, the levels of CD206 protein were significantly higher compared to dim-reared controls (P<0.05). Progressive increases were observed during the post-exposure period, though this was not significant between 3 and 7 days (P>0.05). C, choroid; GCL, ganglion cell layer; INL, inner nuclear layer; IPL, inner plexiform layer; IHC, immunohistochemistry; ONL, outer nuclear layer; OS, outer segments; RPE, retinal pigment epithelium. The trend in ELISA protein levels was significant by ANOVA (P < 0.05); N = 3 for each timepoint.

The spatiotemporal distribution of Il-1β and CD206 markers following light damage was investigated using ELISA and immunhistochemistry (Figs 4–5). Immunoreactivity (IR) for Il-1β protein was absent in dim-reared animals (Fig 4A), but at 24 hrs exposure Il-1β-IR was present among ramified nuclei situated within the ONL and OS (Fig 4B–4D, arrowheads), co-localized with IBA1-IR (Fig 4E–4F, arrowheads). However, outside the vicinity of the ONL Il-1β-IR was not detected in IBA1+ cells (Fig 4F, asterisks). IR for Il1β was not detected at 3 and 7 days post exposure (data not shown). Additionally, we did not detect any IR for CD206 within any of the Il-1β+ positive cells observed (Fig 4G–4H). ELISA for Il-1β protein (Fig 4H) also showcased a strong peak in protein concentration at 24hrs LD (84 pg/mL; P<0.05), and which correlates with the flow cytometry data in Fig 3.

IR for CD206 in dim-reared animals was mostly undetected, except for occasional cells situated in the choroid (Fig 5A, arrows) and ciliary body (Fig 5B, arrows). Following 24hrs LD, increased numbers of CD206+ nuclei were observed in and emerging from the ciliary body and superficial retinal vasculature (Fig 5C–5F, arrows). By 3 days there was an abundance of CD206+ nuclei in the optic nerve head and superficial retinal vasculature (Fig 5G–5I, arrows). This distribution remained mostly unchanged at 7 days (data not shown), although an increase in CD206+ nuclei in the choroid was observed (Fig 5J, arrows). All of the CD206+ cells we detected were IBA1+ (Fig 5K–5L, arrowheads). However, the CD206+/IBA+ cells were morphologically distinct from the ramified Il-1β+/IBA+ cells, and had a different distribution in the retina (*cf*. Fig 3). As with the immunohesotical findings for the Il-1β (Fig 4G–4H), IR for CD206 in the retinas was mutually exclusive and did not show any correlation with labelling Il-1β (Fig 5M–5N). ELISA for CD306 protein (Fig 5O) was mostly consistent the immunohistochemical data. Compared to dim-reared controls, the concentration of CD206 protein steadily increased in the post-exposure period, (1.04 ng/mL) reaching significance at 3 and 7 days post-exposure (1.43 and 1.75 ng/mL respectively; P<0.05).

## Discussion

Our investigation provides a comprehensive profile of M1/M2 activation in a model of light-induced retinal damage, in which we identify distinct temporal and spatial expression patterns of phenotypic markers over a 7 day time course. Up-regulation of M1-associated genes was rapid and transient following light damage, whilst upregulation of M2-related genes was slower and more sustained. We also find that *Il-1β* (M1-associated) and *CD206* (M2-associated) genes are expressed by distinct macrophages subsets over the timecourse of light damage.

While the role of individual cytokines such as Il-6 [35] and Il-1β [36] have been explored previously in models of retinal light stress, the spatiotemporal profile of M1/M2 markers in the retinal macrophage population has not been investigated expansively. While the temporal modulation in M1 and M2 activation profiles has been assessed in retinas following glutamine-induced toxicity, the subsets of macrophage populations has not been fully explored [37]. And, while a study in the CEP-induced AMD model identified increased expression of a number of M1 (*Il-6*, *TNFα*, *Il-1β*) associated-genes in aggregations of subretinal macrophages [17], it did not compare / contrast the timecourse of M1/M2 gene expression profiles. Furthermore, identification of the M2 population in the CEP study was limited to the Arginase 1 marker. In contrast to these studies, our findings provide a more complete picture of the changes in a range of M1 and M2 activation markers in the retinal environment and over time, with respect to light-induced retinal degeneration.

The emergence of retinal M1 and M2 profiles in the present study is consistent with the results of previous investigations [17,37], and with the generally understood patterns of peripheral macrophage polarisation (reviewed in [25,38]). That is, rapid pro-inflammatory responses induced by M1-macrophages are gradually superseded by more regenerative, pro-angiogenic functions of the M2 phenotype. Interestingly, the M1-to-M2 pattern round in the present study is in contrast to the more commonly observed M2-to-M1 transition described in other areas of the CNS (reviewed in [25]).

Although M1 activation likely serves as an initiatory spark, rather than intrinsically deleterious response, recently described functions of ascribed cytokines (such as Il-6, Ccl3, and Tnfα) in augmentation of macrophage activity and photoreceptor death imply a detrimental component of exuberant M1 polarisation in some retinal diseases. In light-stressed Cxc3cr1-/- mice, activated subretinal macrophages up-regulate expression of Il6, promoting their retention in the retinal environment and inducing further photoreceptor death [39]. In the same model, increased Il1β expression by subretinal macrophages is associated with increased photoreceptor apoptosis, which is ameliorated by injections of the endogenous Il1ß inhibitor, Il1ra [36].In other models, Ccl3 expression in activated macrophage/microglia is implicated in both AMD/Stargardt disease (Abca4−/−Rdh8−/− mice) and retinitis pigmentosa (Mertk-/- mice) models [40]. And, in experimental retinal ischemia-reperfusion injury [41], treatment with a TNFα-neutralising antibody reduces in degeneration.

Conversely, there is ample evidence from the CNS suggesting that M2-polarised macrophages promote beneficial functions following injury such as axonal regeneration and vascular repair (reviewed in [25]). In the retina, recruited macrophages which are polarised toward M2 activation via Il-10 signalling are thought to promote retinal ganglion cell survival and regeneration in a model of glutamate-induced toxicity [37]. On the other hand, the pro-angiogenic properties of M2-polarisation are implicated in the pathological vascular conditions such as experimental diabetic retinopathy [42] and CNV [43,44]–where in fact they are deleterious, indicating that broad definitions of ‘deleterious’ M1- vs ‘beneficial’ M2- activation need to be applied prudently [34]. Macrophage polarisation in human retinal donor tissue is sparsely documented, though a small study using AMD donor tissue indicated shifts toward M1 and M2 activation in atrophic and neovascular AMD, respectively, based off ratios of Cxcl10 to Ccl22 expression via qPCR [45].

In the present study, both flow cytometry and immunohistochemistry findings indicate that *Il-1β* (M1) and *CD206* (M2) are expressed by macrophages that have different temporal, spatial and morphological characteristics. Although this is a simplification of M1/M2 activity given these markers only represent a portion of the heterogeneous macrophage population, the data nevertheless support divergent contributions of M1 and M2 responses by macrophage subsets. This echoes the postulations made by Cao and colleagues in their study of AMD donor tissue, of the possible involvement of distinct macrophages subclasses in M1/M2 polarisation in retinal degeneration [45].

The precise origins of these Il-1β and CD206-expressing macrophages—resident, non-resident, or both—and their functional contribution in light damage are beyond the scope of this investigation. That said, the spatiotemporal characteristics of Il-1β and CD206 expressing macrophages in the current study do offer clues to their origin and functional roles in light damage. Our observations of CD206+ macrophages, with their distinctly rounded morphology and association amongst the optic nerve head and ciliary body, are consistent with the recruitment of bone marrow-derived macrophages from the periphery in light-damaged chimeric mice [46]. These observations are also in agreement with chimeric mice subjected to glutamate intoxication, wherein recruited blood-borne macrophages were immunoreactive for M2 markers including TGFβ and Il-10 [37]. In contrast, our data show that Il-1β+ macrophages exhibit traits more closely ascribed to resident microglial cells, including ramified morphology and early emergence within the ONL. It should be noted however that a study by Hu and colleagues showed that cultured bone-marrow derived macrophages up-regulate Il-1β when stimulated with porcine-derived photoreceptor outer segments [36], although the origin of macrophages expressing Il-1β *in vivo* was not assessed. Regardless, we do not discount the capability of non-resident macrophages in also eliciting M1-associated responses, or that M1/M2 fluctuations may be at play in all macrophage populations.

Whether M1/M2 activation represents a broad functional role of resident and non-resident macrophages or only a portion amongst a heterogeneous population, is uncertain. Ccl2 up-regulation is integral to the recruitment macrophages—including blood-borne populations—in the CNS during injury [47,48]. Inhibition of this chemokine pathway has variously yielded protection in models such as light damage [26], Cxc3r1-/- mice [49], CEP-immunised mice [17], laser-induced CNV [50], and autoimmune uveitis [51], or exacerbation of damage in the instance of retinal glutamate intoxication [37]. Based on our findings, we speculate therefore that a portion of recruited blood-borne macrophages encompass an inflammation-resolving M2 phenotype (CD206) which follow the pro-inflammatory M1 response (Il-1β) initially shaped by resident microglia/macrophages during their incursion into the degenerating ONL and subretinal space. The overall benefit of either phenotype however is likely contextual to their abundance within the conglomerate of resident and non-resident macrophages, and the nature of the given disease state. This interpretation is also shared by London and colleagues following their experiments in autoimmune uveitis [51].

## Conclusions

Our findings expand upon previous reports by illustrating the dynamic shift in macrophage polarisation in the light damage model using a broad swathe of established markers. Pro-inflammatory M1 responses appear to dominate the early phase of degeneration, while the chronic post-exposure period is accompanied by persistent up-regulation of M2 markers. The overt role of these phenotypes in light damage is unclear, although the detrimental roles of Il-6, Il-1β, and Ccl3 expressed by macrophages in previous studies certainly suggest a detrimental role of exuberant M1 activation in the photooxidative stress model. However, this does not discount the potential roles of macrophages populations that do not fit the simplified profiles of M1 and M2. We also suggest that resident macrophages/microglia may be initial drivers of potent M1 response in light damage, with the recruitment bone-marrow derived macrophage incorporate a component that is more M2-driven; we emphasise that this is speculative however, and that chimeric experiments are required to verify this in future investigations. Better understanding of the spatiotemporal interplay of macrophage polarisation is an important step in uncovering the functional consequences of their incursion into the retina during disease, and the tailoring of therapeutics toward macrophage subsets which may exert more detrimental activity.
